# Clinical and Immune Responses of Peripheral Chemical Sympathectomy in Enterovirus 71 Infection

**DOI:** 10.3389/fimmu.2021.700903

**Published:** 2021-09-09

**Authors:** Yu-Ting Liao, Huey-Pin Tsai, Shih-Min Wang, Shun-Hua Chen

**Affiliations:** ^1^Department of Pediatrics, College of Medicine, National Cheng Kung University and Hospital, Tainan, Taiwan; ^2^Department of Pathology, National Cheng Kung University Hospital, College of Medicine, National Cheng Kung University, Tainan, Taiwan; ^3^Department of Medical Laboratory Science and Biotechnology, College of Medicine, National Cheng Kung University, Tainan, Taiwan; ^4^Center of Infectious Disease and Signaling Research, National Cheng Kung University, Tainan, Taiwan; ^5^Department of Microbiology & Immunology, College of Medicine, National Cheng Kung University, Tainan, Taiwan

**Keywords:** enterovirus 71, brainstem encephalitis, 6-hydroxydopamine, cytokines, immunophenotypes

## Abstract

The activation of the sympathetic nervous system, release of norepinephrine (NE), and adrenergic receptor signaling participate in and regulate the complicated enterovirus 71 (EV71) brainstem encephalitis (BE). The neurotoxin 6-hydroxydopamine (6-OHDA) selectively ablates sympathetic nerves and markedly depletes NE in innervated organs. Changes in the plasma levels of NE, severity score, cytokine profiles, and percentages of immunophenotype expression in 7-day-old Bltw : CD1 (ICR) mice infected with EV71, with or without 6-OHDA treatment, were compared. The survival rate (76.9%) of EV71-infected and 6-OHDA (30 μg/g)-treated mice was increased significantly. The clinical scores were decreased markedly on days 8-12 in MP4-infected and 6-OHDA-treated mice compared to those without treatment. The results showed that the plasma levels of NE, epinephrine, and dopamine were decreased on days 4–8 after 6-OHDA treatment and at most on day 8. The plasma levels of interleukin (IL)-12p70, tumor necrosis factor, IL-6, and IL-10 did not change significantly after 6-OHDA treatment. Interferon-γ levels decreased evidently on days 4, 6, and 8 after 6-OHDA treatment. The absolute events of CD3^+^CD4^+^, CD3^+^CD8^+^, and CD3^+^NK1.1^+^ cells of peripheral blood mononuclear cells were increased significantly in MP4-infected and 6-OHDA-treated mice compared to those without treatment. In splenocytes, the absolute cells of CD3^−^NK1.1^+^, CD3^+^NK1.1^+^ and CD11b^+^Gr-1^+^ cells of EV71-infected mice were increased significantly after 6-OHDA treatment. These findings suggested that 6-OHDA may be used a probe to explore clinical improvements and immune responses in the complicated EV71 infection. Taken together, peripheral chemical sympathectomy contribute to further understand the immunopathogenesis of EV71 BE with autonomic nervous system dysregulation.

## Introduction

Human enterovirus 71 (EV71) is a positive-sense single-stranded RNA virus that belongs to the family Picornaviridae. Outbreaks of this emerging and life‐threatening pathogen have been reported in many areas in the Asia Pacific (1, 2). The initial outbreak in Taiwan was in 1998, with about 1.5 million infected patients. EV71 infection involves the clinical features of the characteristic cutaneous symptoms, such as hand-foot-mouth disease (HFMD), herpangina, and severe neurological syndromes (1–4). Approximately 30% of hospitalized patients with HFMD with EV71 infection deteriorated owing to severe complications. Brainstem encephalitis (BE) is the major neurological complication in children (1, 2, 5). EV71 BE is categorized into uncomplicated BE, autonomic nervous system (ANS) dysregulation, and pulmonary edema (PE) (3, 4, 6). EV71-infected patients in the ANS dysregulation phase display hypertension, tachycardia, and severe peripheral vasoconstriction. In a previous study, patients with complicated EV71 BE had elevated plasma levels of sympathetic mediators, such as norepinephrine (NE) and epinephrine (EP) (7).

Abundant catecholamines and a strong central nervous system (CNS) inflammatory response aggravate a cytokine storm and pulmonary vascular permeability, resulting in PE of EV71 infection (1, 8). Elevated plasma levels of interleukin (IL)-10, IL-13, and interferon-γ (IFN-γ) were observed in patients with PE. Patients with PE also had lower circulating CD4^+^ and CD8^+^ T cells and natural killer (NK) cells (6, 9–11). Previous studies have demonstrated elevated plasma levels of NE and EP in EV71-infected patients with ANS dysregulation and PE. Both NE and EP enhanced the percentages of infected cells and virus titers in EV71 infection *in vitro* (12). The immune response can exert an influence on the sympathetic nervous system (SNS), and the regulation of immune responses could affect the homeostatic neuronal function (13).

Several experimental approaches have been used to characterize sympathetic innervation and NE release in peripheral tissues. One method has ablated the SNS *via* chemical sympathectomy using 6-hydroxydopamine (6-OHDA) injected peripherally to assess immune function. 6-OHDA selectively ablates sympathetic nerves and markedly depletes NE in innervated organs. 6-OHDA is taken up by NE transporters into the nerve terminals, leading to the destruction of sympathetic nerve terminals, but sparing cell bodies (14). As 6-OHDA does not cross the blood–brain barrier (BBB), the peripheral administration of 6-OHDA does not affect the catecholamine levels in the CNS (15). Chemical sympathectomy with 6-OHDA treatment leads to potential alterations in physiology, for example, decreased arterial blood pressure, increased mesenteric and microcirculatory blood flow, and blood flow to immune organs (16, 17), with a dramatic alteration of cytokine production (18). However, the effects of 6-OHDA on sympathetic dysfunction in the EV71-infected mouse model have not yet been explored. This study investigated the clinical and immune effects of 6-OHDA administration on the changes in clinical severity, mortality, cytokines, and immunophenotype expression in an MP4-infected mouse model.

## Material and Methods

### Cell and Viruses

Human rhabdomyosarcoma (RD; no. 60113) cells were purchased from the Bioresource Collection and Research Center (Hsinchu, Taiwan). The cells were maintained in Dulbecco’s modified Eagle’s medium (#12800-017, Thermo Fisher Scientific) with heat-inactivated 10% fetal bovine serum (FBS; #04-001-1A, Biological Industries). The Virology Laboratory of the National Cheng Kung University Hospital provided an EV71 strain, Taiwan/4643/98. A mouse-adapted EV71 strain, MP4, was prepared according to a previous study (12). Viruses were propagated in RD cells in DMEM supplemented with 2% heat-inactivated FBS.

### Chemical Sympathectomy

6-OHDA hydrochloride (Cat# H4381, Sigma) was dissolved in sterile saline containing 0.02% (w/v) ascorbic acid (vehicle) and was injected intraperitoneally (i.p.). Control mice received injections of an equal volume of the vehicle i.p. To verify the effectiveness of this treatment in depleting catecholamines, plasma was collected and analyzed for the presence of NE, EP, and dopamine (DP).

### Mouse Experiments and Experimental Design

Seven-day-old ICR mice were infected with a 50% lethal dose (LD_50_) of MP4 by i.p. injection. After infection, mock- and MP4-infected mice were administered i.p. with 6-OHDA or an equivalent volume of the vehicle. Their body weights, clinical scores, and survival rates were recorded daily for 14 days. Clinical disease was scored as follows: 0, healthy; 1, ruffled fur and hunchback appearance; 2, wasting; 3, limb weakness; 4, limb paralysis; and 5, moribund and death. All animal experiments and protocols of this study were approved according to the rules of the Animal Protection Act of Taiwan by the Institutional Animal Care and Use Committee of National Cheng Kung University (IACUC #107065) and followed the guidelines established by the Ministry of Science and Technology of Taiwan.

### NE, EP, and DP Measurements

The plasma concentrations of NE, EP, and DP were measured using a commercial immunoassay kit (#BA E-5600, Labor Diagnostika Nord GmbH & Co. KG). NE, EP and DP were extracted by using a cis-diol-specific affinity gel in controls, samples and standards, which was then acylated and derivatized enzymatically. Extracted supernatants were quantified using a competitive enzyme-linked immunosorbent assay (ELISA), according to the manufacturer’s instructions. To ensure the quality of measurements, the controls were included in the assay.

### Cytokine Detection

Cytometric bead array (CBA) kits (BD Pharmingen) were used to detect the presence of IL-12p70, tumor necrosis factor (TNF), monocyte chemoattractant protein-1 (MCP-1), IL-6, IFN-γ, and IL-10 in plasma. Briefly, six populations of beads with distinct fluorescence intensities were coated with cytokine-specific capturing antibodies. The cytokine-captured beads were then mixed with 50-μL specimens and phycoerythrin-conjugated detection antibodies to form sandwich complexes. After incubation, washing, and fluorescence data acquisition, the results were generated using the BD FCAP array software (version 1.0.8).

### Flow Cytometry

Mouse peripheral blood mononuclear cells (PBMCs) were isolated from whole heparin blood using red blood cell (RBC) lysis buffer (#00-4300-54, Thermo Fisher Scientific) and resuspended in staining buffer, containing 2% FBS and 0.1% NaN_3_ in PBS. Spleens were collected and homogenized. Then, the cell suspensions were added lysis buffer to lyse RBCs and resuspended in staining buffer. Cell debris was removed by cell strainer (#352235, Corning). Cells were stained with antibodies purchased from eBioscience (Thermo Fisher Scientific) and BD Pharmingen: BV421-conjugated CD3 antibody (#564008), BV605-conjugated CD4 antibody (#563151), BB515-conjugated CD11b antibody (#564454), APC-conjugated CD8 antibody (#17-0081), PE-Cy7-conjugated CD19 antibody (#25-0193), PerCP-Cy5.5-conjugated Gr-1 antibody (#45-5931), PE-Cy7-conjugated Ly-6C antibody (#25-5932), PE-conjugated NKp46 antibody (#506757), and APC-conjugated NK1.1 antibody (#550267). Data were acquired and analyzed using flow cytometry (BD LSRFortessa) and FlowJo software (version 10; Becton Dickinson Co.).

### Statistical Analyses

Data are presented as the mean ± standard error of the mean (SEM). The Mann–Whitney test and one-way analysis of variance (ANOVA) with the Kruskal–Wallis test were used to analyze the significance. Kaplan–Meier survival curves were analyzed by the log-rank (Mantel–Cox) test. Differences were considered significant at *P* < 0.05. All analyses were performed using GraphPad Prism 5 (GraphPad Software, Inc.).

## Results

### Plasma Levels of NE, EP, and DP in MP4-Infected ICR Mice Without or With 6-OHDA Treatment

**Figure 1:**
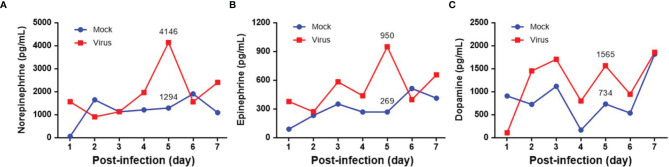
The plasma levels of **(A)** norepinephrine (NE), **(B)** epinephrine (EP) and **(C)** dopamine (DP) in MP4-infected ICR mice. Seven-day-old ICR mice were infected with LD_50_ of MP4 by intraperitoneal (i.p.) injection. Cardiac puncture blood collection was performed, and plasma were separated immediately by centrifugation and stored at -70°C before assay. Plasma samples were pooled from 4 to 5 mice of each data point. Pooled plasma were extracted by using a cis-diol-specific affinity gel, acylated and then derivatized enzymatically. The measurements of NE, EP and DP on extracted samples was achieved by competitive ELISA. Each data point of each group represented the results from a pooled plasma sample.

**Figure 2:**
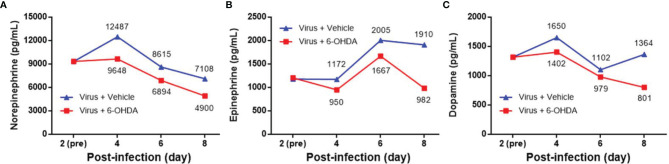
The plasma levels of **(A)** norepinephrine (NE), **(B)** epinephrine (EP) and **(C)** dopamine (DP) after 6-OHDA treatment. Seven-day-old ICR mice were infected with MP4 by i.p. injection. At day 4 after infection, MP4-infected mice were administered i.p. with 30 μg/g/100 μL of 6-OHDA or an equivalent volume of vehicle. After one hour of 6-OHDA delivery at day 4, cardiac puncture blood collection was performed, and plasma were separated immediately by centrifugation and stored at -70°C before assay. Plasma samples were pooled from 4 to 6 mice of each data point. NE, EP, and DA were measured by competitive ELISA. Each data point of each group represented the results from a pooled plasma sample. Pre: pre-6-OHDA injection.

Seven-day-old ICR mice were infected with LD_50_ of MP4 by i.p. injection. Plasma levels of NE, EP, and DP were detected by ELISA. NE increased on day 2 and reached a peak on day 5 after infection (**Figure 1A**). EP increased on day 2, but decreased slightly on day 4. EP reached the peak on day 5, similar to NE (**Figure 1B**). DP increased on day 1 and reached a peak on day 3. The DP levels frequently changed between days 3 and 7. DP dropped on days 4 and 6, but elevated subsequently on days 5 and 7 (**Figure 1C**). The NE and EP levels of the mock group had no significant peak-like virus group (**Figures 1A** **, B**). The DP levels of the mock and virus groups had variable changes day by day. Thus, on day 4 after infection, MP4-infected mice were administered i.p. with 30 μg/g/100 μL 6-OHDA or an equivalent volume of the vehicle (0.9% saline, 0.02% ascorbate). After 1 h of 6-OHDA delivery on day 4, cardiac puncture blood collection was performed, and plasma was collected immediately. Plasma NE, EP, and DP levels were detected by ELISA and compared to their levels before the 6-OHDA injection. NE and DP decreased progressively after 6-OHDA treatment on day 4 (**Figure 2A**). EP increased after 6-OHDA treatment from days 4 to 6, but decreased from days 6 to 8 (**Figure 2B**). The NE, EP, and DA levels in MP4-infected and 6-OHDA-treated mice were lower than those in the MP4-infected and vehicle-treated mice (**Figure 2**). Consequently, 6-OHDA reduced sympathetic overactivation on peripheral tissue and systemic compartments in MP4-infected mice.

### 6-OHDA Treatment in the MP4-Infected Mouse Model

**Figure 3:**
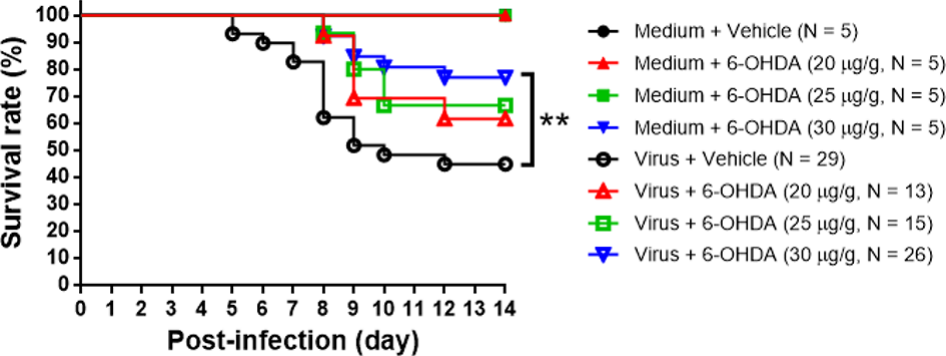
The 6-OHDA treatment in MP4-infected mouse model. Seven-day-old ICR mice were infected with LD_50_ of MP4 by i.p. injection. At day 4, mock-infected and MP4-infected mice (N = 5 for each mock-infected group; N = 13 to 29 for each MP4-infected group) were administered i.p. with 6-OHDA (20, 25 or 30 μg/g in 100 μL) or an equivalent volume of vehicle. Survival rate were recorded until day 14 post-infection. The Kaplan-Meier survival curves were analyzed by the log-rank (Mantel-Cox) test. **P < 0.01.

**Figure 4:**
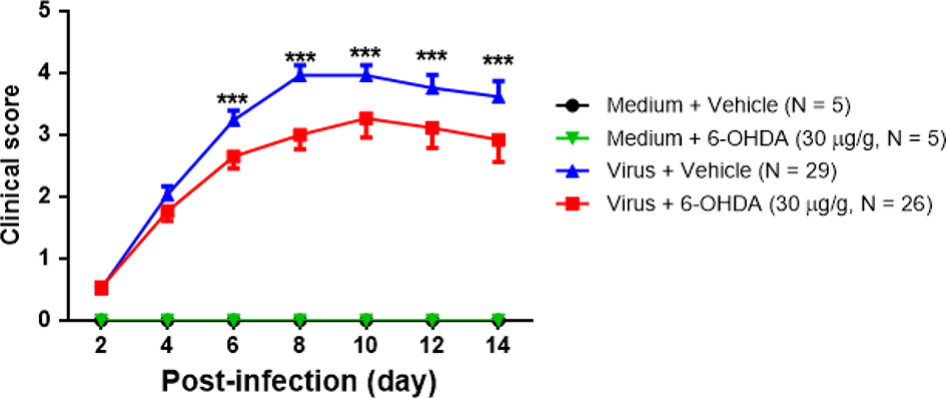
The clinical scores of MP4-infected and 6-OHDA sympathectomized mice. Seven-day-old ICR mice were infected with LD_50_ of MP4 by i.p. injection. At day 4 after infection, mock-infected and MP4-infected mice were administered i.p. with 30 μg/g/100 μL of 6-OHDA or an equivalent volume of vehicle. Clinical scores were recorded every day and until day 14 post-infection. The data shown are the mean ± SEM values and analyzed each group of post-infection day by the One-way ANOVA test. ***P < 0.001.

Seven-day-old ICR mice were infected with LD_50_ of MP4 by i.p. On day 4 after infection, mock- and MP4-infected mice were administered 6-OHDA (20, 25, or 30 μg/g in 100 μL) or an equivalent volume of vehicle i.p. The survival rate of infected mice treated with 20 μg/g 6-OHDA (61.5%) or 25 μg/g 6-OHDA (66.7%) was higher than that of vehicle-treated mice (44.8%). No significant difference was found in 20 μg/g (*P* = 0.204) or 25 μg/g (*P* = 0.110) 6-OHDA-treated mice compared to vehicle-treated mice. The survival rate of infected mice treated with 30 μg/g 6-OHDA (76.9%) was elevated significantly (*P* = 0.009; **Figure 3**). Mock- and MP4-infected mice were administered i.p. with 30 μg/g/100 μL of 6-OHDA or an equivalent volume of the vehicle on day 4 after infection. The clinical scores were recorded every day after infection. Mock-infected mice with or without 6-OHDA treatment were healthy and sacrificed until day 14. The clinical scores began to elevate on day 2 and reached the top on day 8. Most mice died from days 6 to 9, and the clinical scores were reduced gradually from days 8 to 14. The clinical scores of MP4-infected and 6-OHDA-treated mice (day 8: 3.02 ± 0.16, day 10: 2.91 ± 0.23, and day 12: 2.31 ± 0.20) were markedly lower than MP4-infected and vehicle-treated mice (day 8: 3.87 ± 0.16, day 10: 3.40 ± 0.20, and day 12: 3.17 ± 0.30) on days 8–12 (**Figure 4**).

### Cytokine Expression in MP4-Infected and 6-OHDA-Treated Mice

**Figure 5:**
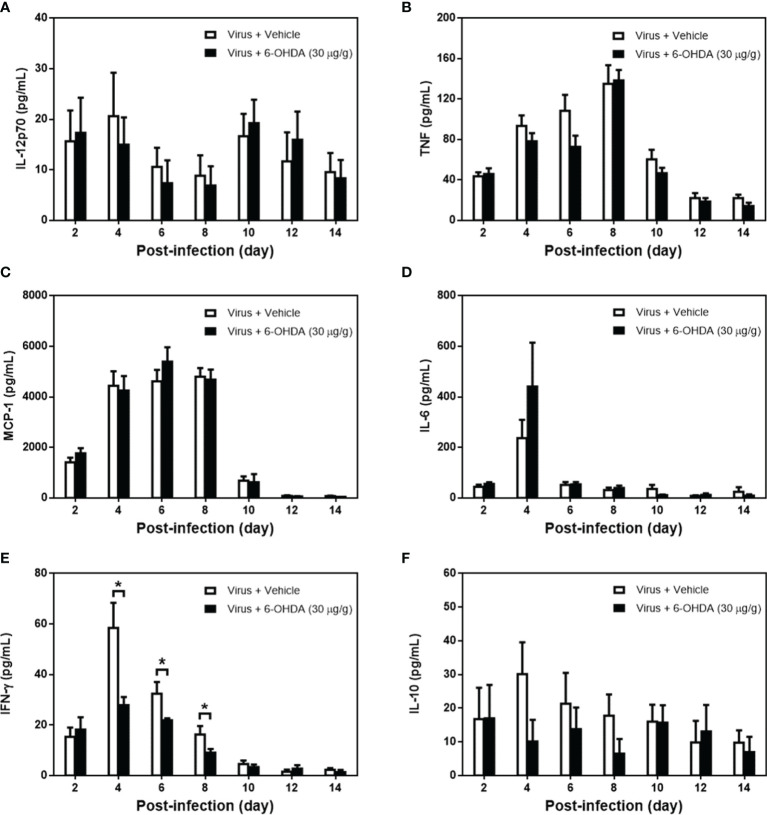
The cytokine expression in MP4-infected and 6-OHDA-treated mice. Seven-day-old ICR mice were infected with MP4 by i.p. injection. At day 4 after infection, MP4-infected mice were administered i.p. with 30 μg/g/100 μL of 6-OHDA or an equivalent volume of vehicle. After one hour of 6-OHDA delivery at day 4, cardiac puncture blood collection was performed, and plasma were separated immediately by centrifugation and stored at -70°C before assay. Plasma were collected at 7 time points from 4 to 12 mice per bar. **(A)** IL-12p70, **(B)** TNF, **(C)** MCP-1, **(D)** IL-6, **(E)** IFN-γ and **(F)** IL-10 were detected by using CBA kits. The data shown are the mean ± SEM values and analyzed each post-infection day by Mann-Whitneytest. *P < 0.05.

After 1 h of 6-OHDA delivery on day 4, cardiac puncture blood collection was performed, and plasma was separated immediately. Plasma was collected to measure IL-12p70, TNF, MCP-1, IL-6, IFN-γ, and IL-10 levels. After infection, the levels of IL-12p70 peaked on days 4 and 10. IL-12p70 diminished on day 8, whereas the clinical score reached the top (**Figure 5A**). The levels of IL-12p70 had no significant change between 6-OHDA-treated mice (day 4: 14.97 ± 5.40, day 6: 7.36 ± 4.54, and day 8: 6.90 ± 3.80) and vehicle-treated mice (day 4: 20.60 ± 8.62, day 6: 10.59 ± 3.77, and day 8: 8.85 ± 4.04). The TNF and MCP-1 levels increased on day 2 and reached a peak on day 8. Then, TNF and MCP-1 were reduced gradually after days 10–14. The tendencies of TNF and MCP-1 were similar to the changes in clinical scores (**Figures 5B** **, C**). On day 6, the TNF level was lower in 6-OHDA treatment (day 4: 78.26 ± 8.18 and day 6: 72.80 ± 11.05), but showed no statistical difference between vehicle treatment (day 4: 93.46 ± 10.52 and day 6: 108.53 ± 15.66; **Figure 5C**). The IL-6 levels peaked on day 4, but dropped drastically on day 6 (**Figure 5D**). IFN-γ peaked on day 4, but decreased progressively from days 6 to 14 (**Figure 5E**). On days 4 and 8, IFN-γ expression was reduced significantly with 6-OHDA treatment (day 4: 27.89 ± 3.23, *P* = 0.016; day 6: 21.94 ± 0.73, *P* = 0.029; and day 8: 9.06 ± 1.45, *P* = 0.042) compared to the vehicle group (day 4: 58.50 ± 9.87, day 6: 32.39 ± 4.64, and day 8: 16.30 ± 3.35). The IFN-γ and IL-6 levels continued to decrease when the clinical scores were increased. The IL-10 level elevated from day 2 and was expressed constantly until day 14, but had no significant change between 6-OHDA-treated mice (day 4: 10.10 ± 6.41, day 6: 13.75 ± 6.45, and day 8: 6.53 ± 4.33) and vehicle-treated mice (day 4: 30.17 ± 9.41, day 6: 21.34 ± 9.17, and day 8: 17.79 ± 6.28; **Figure 5F**).

### Cell Populations of PBMCs and Splenocytes in MP4-Infected and 6-OHDA-Treated Mice

**Figure 6:**
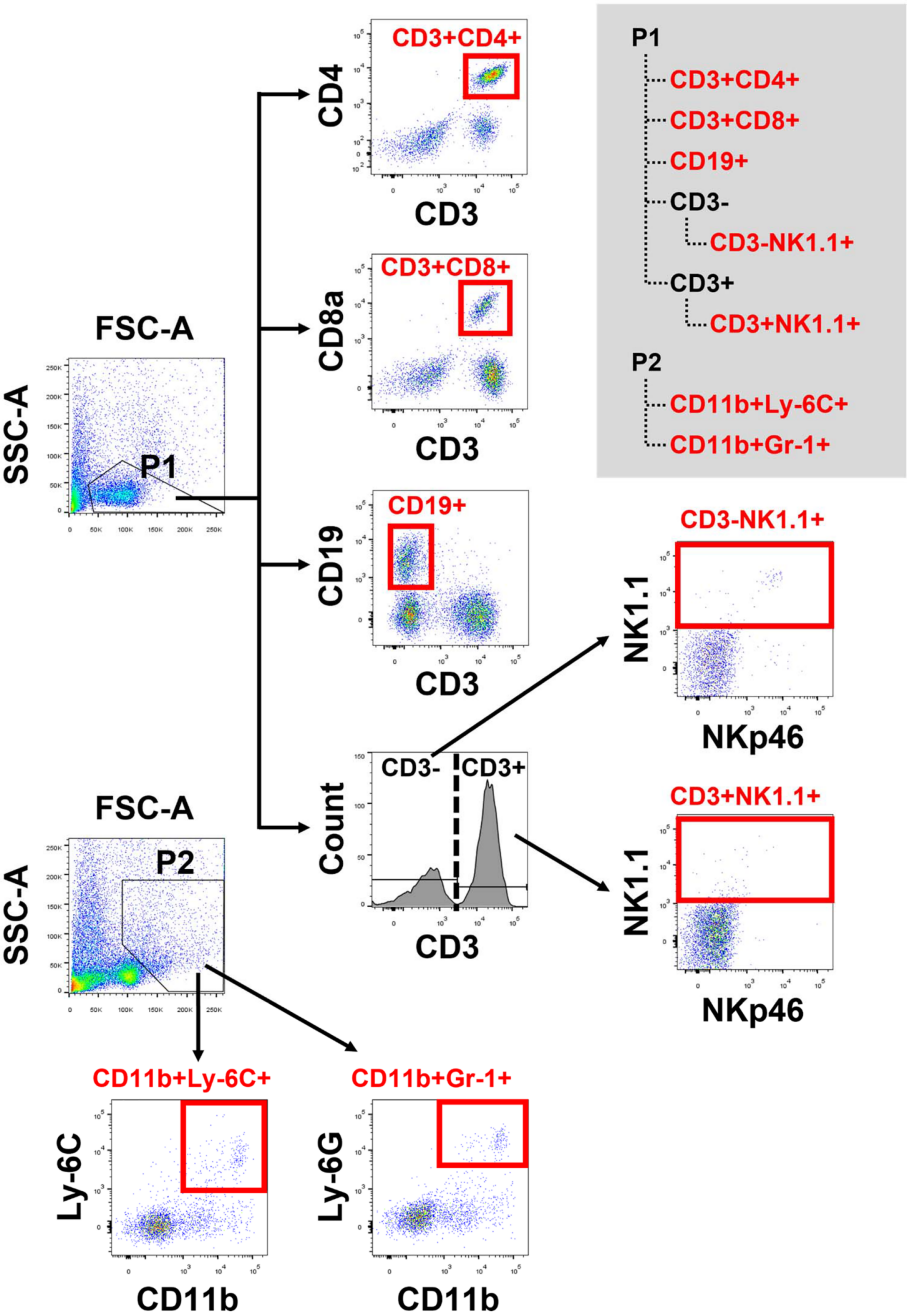
The flow cytometric panel for analyzing mouse PBMCs and splenocytes. Gating strategy to identify lymphocyte subsets from mouse PBMCs and splencytes. P1 and P2 were gated in FSC-A/SSC-A dot plot. From P1, CD3^+^CD4^+^ and CD3^+^ CD8^+^ cells were gated on CD3, CD4 and CD8. CD19^+^ cells were gated on CD3 and CD19. CD3^-^NK1.1^+^ and CD3^+^NK1.1^+^ cells, within CD3^-^ and CD3^+^ cells, respectively, were defined by NK1.1 and NKp46. From P2, CD11b^+^Ly-6C^+^ and CD11b^+^Gr-1^+^ cells were gated on CD11b, Ly-6C and Gr-1.

Cell populations of PBMCs and splenocytes were collected and analyzed on day 6 after infection (**Figure 6**). The absolute events of CD3^+^CD4^+^, CD3^+^CD8^+^, CD19^+^, CD3^−^NK1.1^+^, and CD3^+^NK1.1^+^ cells of PBMCs and splenocytes were lower after infection. However, the higher percentages of CD11b^+^Ly-6C^+^ and CD11b+Gr-1^+^ cells were found in MP4-infected mice (**Tables 1**, **2**). The absolute events of CD3^+^CD4^+^, CD3^+^CD8^+^, and CD3^+^NK1.1^+^ cells of PBMCs were elevated significantly in MP4-infected and 6-OHDA-treated mice compared to MP4-infected and vehicle-treated mice (**Table 1**). The percentages of CD3^+^CD8^+^ cells of PBMCs were higher in MP4-infected and 6-OHDA-treated mice than in medium- and vehicle-treated mice (**Table 1**). In splenocytes, the absolute events of CD3^−^NK1.1^+^, CD3^+^NK1.1^+^ and CD11b^+^Gr-1^+^ cells of MP4-infected mice were increased significantly after 6-OHDA treatment (**Table 2**). Moreover, the percentages of CD3^-^NK1.1^+^, CD11b^+^Ly-6C^+^ and CD11b+Gr-1^+^ of splenocytes were higher in MP4-infected and 6-OHDA-treated mice than in medium- and vehicle-treated mice (**Table 2**). Therefore, cytotoxic T, NK, and NKT cells may contribute to infection after 6-OHDA treatment.

**Table 1:** Cell populations of PBMCs in MP4-infected and 6-OHDA-treated mice at day 6 post-infection.

	Mouse PBMCs						
	Medium + Vehicle (N = 5)		Virus + Vehicle (N = 5)		Virus + 6-OHDA (N = 5)		
	Absolute events (x 10^3^ cell/mL)	%	Absolute events (x 10^3^ cell/mL)	%	Absolute events (x 10^3^ cell/mL)	%	
**CD3^+^CD4^+^**	610.27 (157.35)	20.8 (3.90)	92.54 (19.44)^a^	32.58 (6.20)	574.86 (184.40)^c^	42.60 (8.75)	
**CD3^+^CD8^+^**	206.38 (73.15)	5.81 (0.79)	42.75 (15.67)	13.56 (1.79)^a^	178.51 (48.79)^c^	13.52 (1.14)^d^	
**CD19^+^**	224.33 (68.95)	16.75 (4.95)	12.65 (3.74)^b^	17.15 (4.30)	73.69 (28.88)	19.26 (0.96)	
**CD3^-^NK1.1^+^**	68.32 (12.86)	3.54 (1.13)	8.45 (4.38)^a^	6.28 (2.80)	38.47 (15.87)	7.62 (3.28)	
**CD3^+^NK1.1^+^**	17.87 (2.61)	3.47 (0.69)	7.39 (3.49)	4.76 (1.70)	32.40 (10.36)^c^	8.75 (5.58)	
**CD11b^+^Ly-6C^+^**	77.95 (15.54)	11.24 (2.80)	89.04 (23.56)	52.64 (8.37)^b^	106.48 (26.89)	38.48 (4.08)	
**CD11b^+^Gr-1^+^**	73.54 (10.61)	11.99 (4.27)	78.91 (25.84)	46.40 (9.94)^a^	94.51 (24.24)	34.00 (3.23)	

^a^P < 0.05, for the difference between Medium + Vehicle treated mice and Virus + Vehicle treated mice; ^b^P < 0.01, for the difference between Medium + Vehicle treated mice and Virus + Vehicle treated mice; ^c^P < 0.05, for the difference between Virus + Vehicle treated mice and Virus + 6-OHDA treated mice; ^d^P < 0.05, for the difference between Medium + Vehicle treated mice and Virus + 6-OHDA treated mice. The data shown are the mean (SEM) values and analyzed by Kruskal-Wallis test.

**Table 2:** Cell populations of splenocytes in MP4-infected and 6-OHDA-treated mice at day 6 post-infection.

	Mouse Splenocytes					
	Medium + Vehicle (N = 5)		Virus + Vehicle (N = 4)^$^		Virus + 6-OHDA (N = 5)	
	Absolute events (x 10^3^ cell/mL)	%	Absolute events (x 10^3^ cell/mL)	%	Absolute events (x 10^3^ cell/mL)	%
**CD3^+^CD4^+^**	181.54 (22.26)	8.49 (0.65)	31.22 (9.58)^a^	5.90 (1.58)	119.15 (13.93)	5.99 (0.42)
**CD3^+^CD8^+^**	52.59 (4.49)	2.52 (0.28)	12.44 (3.52)^a^	2.30 (0.48)	41.09 (3.51)	2.16 (0.30)
**CD19^+^**	749.47 (153.44)	54.36 (2.45)	55.30 (14.64)^a^	25.70 (2.30)^a^	344.56 (84.88)	33.04 (4.26)
**CD3^-^NK1.1^+^**	19.61 (2.13)	1.03 (0.07)	8.04 (1.18)	1.69 (0.21)	50.90 (11.68)^c^	2.61 (0.38)^e^
**CD3^+^NK1.1^+^**	5.95 (1.00)	2.18 (0.34)	3.49 (0.76)	7.22 (0.88)^b^	13.57 (2.68)^d^	6.84 (1.07)^f^
**CD11b^+^Ly-6C^+^**	82.60 (11.34)	14.96 (1.88)	124.21 (36.68)	34.75 (2.13)^b^	422.40 (74.95)^f^	35.74 (1.55)^f^
**CD11b^+^Gr-1^+^**	47.87 (6.88)	8.68 (1.15)	52.82 (15.44)	14.88 (1.10)	191.16 (33.67)^d,f^	16.24 (0.99)^f^

^a^P < 0.01, for the difference between Medium + Vehicle treated mice and Virus + Vehicle treated mice; ^b^P < 0.05, for the difference between Medium + Vehicle treated mice and Virus + Vehicle treated mice; ^c^P < 0.01, for the difference between Virus + Vehicle treated mice and Virus + 6-OHDA treated mice; ^d^P < 0.05, for the difference between Virus + Vehicle treated mice and Virus + 6-OHDA treated mice; ^e^P < 0.01, for the difference between Medium + Vehicle treated mice and Virus + 6-OHDA treated mice; ^f^P < 0.05, for the difference between Medium + Vehicle treated mice and Virus + 6-OHDA treated mice. ^$^ 1 missing data was due to analysis failure. The data shown are the mean (SEM) values and analyzed by Kruskal-Wallis test.

## Discussion

EP is an important mediator involved in ANS dysregulation and PE in critical patients with EV71 infection (7, 12). At the same time, this naturally occurring neurotransmitter is endogenously overreleased in these patients (12). Stress-induced hormones can alter inflammatory responses to tissue injury; however, the precise mechanism by which EP influences immune and inflammatory responses is not well defined. 6-OHDA selectively destroys peripheral noradrenergic fibers of the SNS. More importantly, 6-OHDA modulates a variety of immune parameters, including cell-mediated responses (19), cytokine production (17), and lymphocyte trafficking and proliferation (20, 21). This study demonstrated that catecholamine levels increased in the plasma of EV71-infected mice and then decreased after 6-OHDA administration. Also, mortality and the clinical score were decreased and improved, respectively, in MP4-infected mice after 6-OHDA administration. The roles of NE, EP, and DA in the sympathetic overactivity of severe EV71 infection in light of further correlated evidence of ANS dysfunction complications.

Indeed, the levels of proinflammatory cytokines and chemokines, such as IL-6, IFN-γ, IP-10, and monokine induced by gamma interferon (MIG), were elevated in the plasma or cerebrospinal fluid of patients with EV71 BE with PE (8). IFN-*γ* plays a cardinal role in the pathogenesis of EV71 BE with PE in both systemic and CNS compartments of patients (9, 11). Plasma levels of IFN-γ, but not other cytokines, were decreased significantly in EV71-infected mice after 6-OHDA administration. Willemze et al. showed the effect of sympathetic denervation on mucosal innate immune responses using 6-OHDA and found that IFN-γ in colon homogenates was normalized for total protein levels (21). ThyagaRajan et al. also reported reductions in sympathetic noradrenaline innervation and NE levels in the spleen of female F344 rats, accompanied by significant reductions in NK cell activity, IL-2 and IFN-γ production, and T- and B-cell proliferation (22). Our findings supported the notion that 6-OHDA administration in MP4-infected mice possessed anti-inflammatory property and reduced the key cytokine, IFN-γ, in the pathogenesis of ANS dysregulation of EV71 infections.

In this study, the absolute cell counts of CD3^+^CD4^+^, CD3^+^CD8^+^, and CD3^−^NK1.1^+^ in PBMCs were decreased in EV71-infected mice. However, CD3^+^CD4^+^, CD3^+^CD8^+^, and CD3^+^NK1.1^+^ in PBMCs were increased after 6-OHDA administration. In a previous study, patients with severe complications, EV71-associated PE, also had lower circulating CD4^+^ and CD8^+^ T and NK cells in the blood (9). Liao et al. demonstrated that NE and EP treatment increased the percentages of EV71-infected cells in THP-1 and Jurkat cells, respectively. In contrast, α- and β-blockers decreased the percentages of EV71-infected cells with NE or EP treatment (12). EV71 neurological complications can severely affect the increased levels of NE, hypersympathetic innervation, and changes in immune cell frequencies. Further, the absolute counts of CD3^+^CD4^+^, CD3^+^CD8^+^, and CD19^+^ were decreased in the spleen of mice after MP4 infection. In contrast, after 6-OHDA administration, the absolute cell counts and percentages of CD3^−^NK1.1^+^ and the absolute cell counts of CD3^+^NK1.1^+^ were increased in the spleen. The splenic parenchyma is rich in sympathetic innervation expressing both synaptophysin and tyrosine hydroxylase, displaying a unique, complex, and panicle-like neuronal architecture (23). Qi et al. reported that the SNS was involved in the loss of spleen B cells and bone marrow early B cells caused by the H9N2 avian influenza virus. 6-OHDA treatment could rescue the loss of B cells in the spleen and bone marrow (24). However, under EV71 infection, CD19^+^ cells showed no significant change in both PBMCs and splenocytes after 6-OHDA treatment. Monteiro et al. found that low doses of NE stimulation of splenocytes mainly affected the neutrophil population, promoting an increase in both frequency and numbers. The interruption of the sympathetic communication to the spleen by ablating the splenic nerve resulted in reduced frequencies and numbers of neutrophils in the spleen (25). These findings supported the notion that 6-OHDA administration in MP4-infected mice may block the effects of catecholamine, EP, and NE on PBMCs and splenocytes. The sympathetic innervation of lymphoid tissues provides a pathway for products of the nervous system to modulate immune function.

Peripheral chemical sympathectomy in adapted virus infected mouse model to explore the immunopathogenesis of EV71 infection with ANS dysregulation was successfully in this study. Shih and coworkers also testified that immunodeficient neonatal mouse models can be infected with EV71 clinical isolates. Further, crossbreeding between SCARB2 transgenic and stat1 knockout mice may generate a more sensitive and user-friendly hybrid mouse model (26). Recently, a study showed that TLR7 immune modulators in EV71 infections of immunocompetent mouse 129 model, may be another possible alternative (27).

In conclusion, SNS mediators, EP and NE, dysregulate the clinical severity and cytokine profile, constrain the innate immune cell reactivity of host responses, and play critical roles in ANS dysregulation in EV71 infection.

## Data Availability Statement

The raw data supporting the conclusions of this article will be made available by the authors, without undue reservation.

## Ethics Statement

The animal study was reviewed and approved by Affidavit of Approval of Animal Use Protocol National Cheng Kung University.

## Author Contributions

SM-W designed research and revised the manuscript. SM-W obtained funding. YT-L, HP-T, SH-C, and SM-W implemented research. YT-L and SM-W interpreted data, analyzed statistics, and drafted the manuscript. All authors contributed to the article and approved the submitted version.

## Funding

This study was supported by grant (MOST 107-2314-B-006-045, MOST 109-2327-B-006-009) Ministry of Science and Technology, Taiwan.

## Conflict of Interest

The authors declare that the research was conducted in the absence of any commercial or financial relationships that could be construed as a potential conflict of interest.

## Publisher’s Note

All claims expressed in this article are solely those of the authors and do not necessarily represent those of their affiliated organizations, or those of the publisher, the editors and the reviewers. Any product that may be evaluated in this article, or claim that may be made by its manufacturer, is not guaranteed or endorsed by the publisher.
